# Mechanistic Insights and Rational Design of a Versatile Surface with Cells/Bacteria Recognition Capability via Orientated Fusion Peptides

**DOI:** 10.1002/advs.201801827

**Published:** 2019-03-04

**Authors:** Lin Wang, Junjian Chen, Xiangze Zeng, Peter Pak‐Hang Cheung, Xiaoyan Zheng, Liangxu Xie, Xuetao Shi, Li Ren, Xuhui Huang, Yingjun Wang

**Affiliations:** ^1^ National Engineering Research Center for Tissue Restoration and Reconstruction South China University of Technology Guangzhou 510006 P. R. China; ^2^ Department of Chemistry Hong Kong Branch of Chinese National Engineering Research Center for Tissue Restoration & Reconstruction Hong Kong University of Science and Technology Clear Water Bay Kowloon Hong Kong China; ^3^ School of Biomedical Science and Engineering South China University of Technology Guangzhou 510006 P. R. China

**Keywords:** antibacterial activity, fusion peptides, molecular dynamics simulation, solvent accessible surface area, surface modification

## Abstract

Hospital‐acquired infection causes many deaths worldwide and calls for the urgent need for antibacterial biomaterials used in clinic that can selectively kill harmful bacteria. The present study rationally designs fusion peptides capable of undergoing 2D self‐assembly on the poly(methyl methacrylate) surface to form a smart surface, which can maintain a desirable orientation via electrostatic interactions. The in vitro assay shows that the smart surface can recognize bacteria to exert antibacterial activity and is nontoxic toward mouse bone mesenchymal stem cells. Excitingly, the smart surface can distinguish different bacterial strains. This selective feature, from being broad‐spectrum to being highly selective against *S. aureus*, can be altered by varying the number of amino acids in the recognition sequences. By all‐atom molecular dynamics simulations, it is also found that the recognition sequence in the peptide is critical for the selectivity toward specific bacterial strains, in which a less accessible surface area for the bacteria in the antimicrobial peptide sequence is responsible for such selectivity. Finally, the smart surface can inhibit *S. aureus* infection in vivo with much more rapid tissue‐healing compared to the control.

Bacterial infection remains a serious global problem,^1^, ^2^ and biomaterial‐associated infection accounts for ≈45% of all hospital‐acquired infections.^3^ Bacteria can form biofilm on the biomaterial surface, such as catheters,^4^, ^5^ fracture fixation devices,^6^ stents,^7^ and ophthalmology.^8^ The biofilm can protect the bacteria from antibiotics and host immune system.^9^, ^10^, ^11^ Hence, infection can only be prevented by removing the infected biomaterial that potentially leads to surgical failure, poor disease outcome, and even death.^12^

An efficient method to inhibit infection of the biomaterial is to modify its surface with antibacterial agents containing silver, antibacterial polymers, antibiotics, etc.^13^, ^14^, ^15^, ^16^, ^17^ Among these, the antimicrobial peptides (AMPs) hold great potential due to their high antibacterial activity, low drug‐resistance, and specific metabolic pathways.^18^ Until now, more than 2300 AMPs have been identified from natural sources.^19^ In addition, many synthetic AMPs, especially those with short sequence, have been rationally designed. For example, HHC36 (Lys‐Arg‐Trp‐Trp‐Lys‐Trp‐Trp‐Arg‐Arg),^20^ has been shown to confer biomaterial surface with high antibacterial activity both in vitro and in vivo.^21^, ^22^, ^23^

Unfortunately, the toxicity toward mammalian cells limits the use of most antibacterial surfaces, including those that are AMPs‐based.^21^, ^24^, ^25^, ^26^ Such toxicity prohibits cell adhesion that is critical to the healing process, hence increasing the risks of infection.^27^ To address this issue, AMPs are conjugated with hemocompatible/biocompatible polymers to reduce cytotoxicity. These polymers include amphiphilic poly(acrylic acid‐*b*‐styrene) block copolymer,^28^ four‐arm star glycopolymers,^29^ and polyphosphoesters.^30^ And other biocompatible AMPs‐mimic polymers are also designed, such as triblock copolymer of poly[ethylene oxide]–poly[propylene oxide]–poly[ethylene oxide] (PEO–PPO–PEO)^31^ and the nylon‐3 derivative copolymers.^17^, ^32^, ^33^ These modified AMPs and AMPs‐mimic polymers can be used in clinic to reduce the toxicity of antibacterial surfaces.^9^, ^34^, ^35^ In particularly, some of these molecules are sensitive and display bioactivities to specific microenvironment.^36^, ^37^, ^38^, ^39^ However, these methods suffer the following drawbacks: 1) Many polymers cannot be degraded in vivo or exhibit unknown metabolic pathways, causing inflammation and increasing the risk of adverse reactions.^9^ 2) The preparation processes of these surfaces often contain many steps which include heating and organic solvent that can impact the bioactivity of the molecules and make it difficult to control the integration density of the AMPs on the surface.^40^, ^41^ In our previous study, we have removed the polymers and prepare versatile antibacterial surfaces by mixing the AMPs (HHC36) with the biocompatible peptide RGD (Arg‐Gly‐Asp) to enhance the biocompatibility and cell adhesion of the biomaterials.^26^ But without the polymers, the ability of the surface to recognize the microenvironment is lost, which will cancel out different bioactivities of the peptides on the surface. Concretely, a decrease in the cytotoxicity of the surface also reduces its antibacterial activity. This method also contains complicated covalent linkages to regulate and optimize the orientation of the peptides on the surface. Until now, there is lack of simple and efficient methods to prepare versatile surface that exhibits specific bioactivity to different microenvironments with low cytotoxicity.

In the present study, we report a convenient 2D self‐assembly method to prepare a versatile surface with cell/bacteria recognition capability based on the rationally designed fusion peptide (**Figure** **1**a). The orientation of the fusion peptide on the surface is crucial for the versatility of the bioactivity, although it is difficult to control by the physical interaction in 2D self‐assembly. Theoretically, electrostatic interactions via opposite electrical property can keep the peptide at regular orientation on an electriferous surface. To optimize the orientation of the surface peptide, we employed HHC36 as the N‐terminal (abbreviated as AMP sequence), and RGD as the C‐terminal (abbreviated as RGD sequence) with opposite zeta potential, which was +14.1 eV for the N‐terminal, and was −9.9 eV for the C‐terminal (Table S1, Supporting Information). Also, we inserted the recognition sequences Gly‐Val or Gly‐Pro‐Leu‐Gly‐Val, which could shield parts of the underlying molecular bioactivity and be bioactive to bacterial infection, since it was only cleaved by bacteria‐secreted protease, i.e., gelatinase,^42^, ^43^ between the AMP and RGD sequences. The sequences of the two fusion peptides were Lys‐Arg‐Trp‐Trp‐Lys‐Trp‐Trp‐Arg‐Arg‐Gly‐Val‐Arg‐Gly‐Asp (with 14 amino acids, abbreviated as KD14) and Lys‐Arg‐Trp‐Trp‐Lys‐Trp‐Trp‐Arg‐Arg‐((Gly)‐Pro‐Leu‐Gly‐)Val‐Arg‐Gly‐Asp (with 17 amino acids, abbreviated as KD17), respectively (Figure 1a).

**Figure 1:**
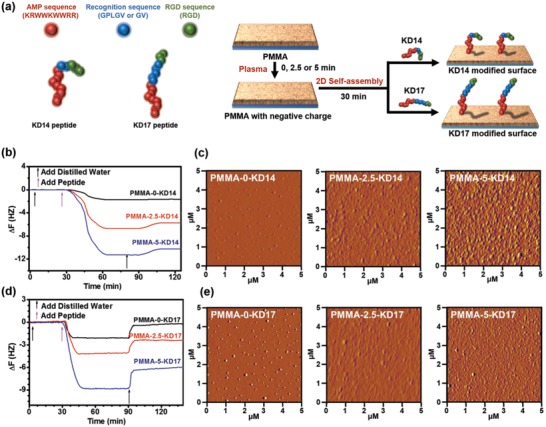
a) The sequence of KD14 and KD17, and the schematic diagram of the 2D self‐assembly of the indicated peptides on the surfaces. The AMP sequence (KRWWKWWRR) was marked by red, the recognition sequence (GV or GPLGV) was marked by blue, and the RGD sequence (RGD) was marked by green. b) The QCM‐D results of the 2D self‐assembly of KD14 on different PMMA surfaces. c) The atomic force microscope (AFM) images of the indicated surfaces with KD14. d) The QCM‐D results of the 2D self‐assembly of KD17 on different PMMA surfaces. e) The AFM images of the indicated surfaces with KD17. See Sections 3–6 in the Supporting Information for the details of the preparation process and assay.

Results from high‐performance liquid chromatography (HPLC) and high‐resolution mass spectrometry (HRMS) demonstrated that the synthesized fusion peptides had high purities (Figures S1 and S2, Supporting Information), and the designed peptides could be cleaved at the Gly‐Val site by gelatinase (details in Figures S3 and S4, Supporting Information). These two fusion peptides had positive zeta potentials, while KD14 had the zeta potential of +5.8 eV, and KD17 had the zeta potential of +5.1 eV (Table S1, Supporting Information). We found that the antibacterial activity of the AMP sequence was not affected by fusing the RGD sequence with recognition sequence. The two fusion peptides showed similar antibacterial activities to the HHC36 against both *S. aureus* and *E. coli* in solution (Figure S5, Supporting Information).

To integrate the peptides on the surface and establish their regular orientation, we chose the electronegative poly(methyl methacrylate) (PMMA) that has a wide application in ophthalmological and orthopedic clinics prone to common bacterial infection.^44^, ^45^, ^46^, ^47^, ^48^ We employed oxygen plasma treatment to enhance the electronegativity of the PMMA substrates. By controlling the treatment time, we could generate the surfaces with different negative zeta potentials. Before treatment, the zeta potential of PMMA‐0 min (pristine PMMA) surface was −20.1 eV (Figure S6, Supporting Information). The negative zeta potential of the surface increased with the treatment time. After being treated for 2.5 min (PMMA‐2.5 min) and 5 min (PMMA‐5 min), the zeta potentials of the surfaces were −32.3 and −55.7 eV, respectively (Figure S6, Supporting Information).

Both KD14 and KD17 could self‐assemble on the PMMA surfaces, and the integration density increased together with the negative zeta potential. We abbreviated the name of the surfaces with different zeta potentials and peptides as PMMA‐*m*‐n, in which m represented the plasma treatment time of 0, 2.5, or 5 min, and n represented different peptides (Table S2, Supporting Information). The quartz crystal microbalance with dissipation (QCM‐D) results showed that after the 2D self‐assembly of the KD14, the mass of the substrate increased, which was proportional to the negative zeta potential of the surface (Figure 1b). Based on Q‐tools calculations, the densities of the KD14 on PMMA‐0 min, PMMA‐2.5 min, and PMMA‐5 min were 46.5, 136.1, and 238.5 ng cm^−2^, respectively (Table S3, Supporting Information). The atomic force microscope (AFM) results showed that the morphology of the surface changed evidently with the integration of KD14, and PMMA‐5‐KD14 had evident bulges compared to others (Figure 1c). Meanwhile, the QCM‐D assay showed that the densities of KD17 on PMMA‐0 min, PMMA‐2.5 min, and PMMA‐5 min were 5.0, 62.2, and 136.6 ng cm^−2^, respectively, and they were also proportional to the negative zeta potential of the surface (Figure 1d). Compared to KD14, the density of KD17 was lower on the surface with the same zeta potential. The AFM results also showed that compared to PMMA‐0‐KD17 and PMMA‐2.5‐KD17, PMMA‐5‐KD17 had evident bulges (Figure 1e). In addition, the fusion peptides affected the hydrophobicity of the PMMA surface. After being integrated with the fusion peptides, the contact angle of PMMA‐5‐KD14 was 56.5° and PMMA‐5‐KD17 was 54.2°, which was lower than that of PMMA substrate (71.8°) and higher than that of PMMA‐5 min (36.9°) (Figure S7, Supporting Information), respectively.

Further binding free energies computed from the mole‐cular mechanics Poisson–Boltzmann surface area (MM/PBSA) method based on all‐atom molecular dynamics (MD) simulations^49^ illustrated that both fusion peptides on the surface adopted a specific orientation, in which the AMP sequence was more likely to bind to the surface than the RGD sequence, as the AMP sequence had a much stronger binding affinity than that of the RGD sequence. To simulate the PMMA substrates with different zeta potentials, we used PMMA molecules with 10 units to prepare the substrate (Figure S8, Supporting Information), and added 5, 15, or 25 negative charges on the substrates in the area of 8 nm × 8 nm, which were abbreviated as 5_PMMA, 15_PMMA, and 25_PMMA, respectively (for details, see the Supporting Information). The representative MD snapshots demonstrated that the peptide could adsorb on the substrate (Figure S9, Supporting Information). The time evolutions of the distance between the center of mass (COM) of peptide and the COM of PMMA substrate along *Z* axis (Figure S10, Supporting Information), and the contact area between the peptide and the substrate (Figure S11, Supporting Information) clearly indicated that our MD simulations reached convergence after 20 ns. The results also showed that the binding free energy of the 5_PMMA to the AMP sequence in KD14 was −618.5 kJ mol^−1^ (Figure S12a, Supporting Information), and the binding free energies for 15_PMMA and 25_PMMA were −1998.0 and −2586.0 kJ mol^−1^ (Figure S12a, Supporting Information). The binding free energies of the RGD sequence to 5_PMMA, 15_PMMA, 25_PMMA were +32.7, +154.3, and +298.3 kJ mol^−1^, respectively (Figure S12b, Supporting Information), indicating a smaller binding affinity than AMP sequence. We further demonstrated that the binding free energy between the AMP sequence in the KD14 and the surface was mainly contributed by the electrostatic interactions, and the contributions from the electrostatic energies in 5_PMMA, 15_PMMA, and 25_PMMA were −676.4, −2638.0, and −3269.0 kJ mol^−1^, respectively (Figure S12c, Supporting Information). Similar to KD14, the MD simulation results showed that 5_PMMA, 15_PMMA, and 25_PMMA were able to attract the AMP sequence in KD17 with the binding free energies of −390.5, −1824.0, and −2686.0 kJ mol^−1^ (Figure S13a, Supporting Information). And the surface exhibited smaller binding affinity to RGD sequence in KD17 with the binding free energies of −66.3, 35.0, and −30.8 kJ mol^−1^, respectively (Figure S13b, Supporting Information). Moreover, the binding free energy between the AMP sequence in the KD17 and the surface was also mainly contributed by the electrostatic interactions, and the contributions were −402.2, −2315.0, and −3203.0 kJ mol^−1^ on 5_PMMA, 15_PMMA, and 25_PMMA, respectively (Figure S13c, Supporting Information).

We found that both KD14‐ and KD17‐modified surfaces could recognize the mBMSCs cells to exhibit improved biocompatibility due to the upper RGD sequence in this theoretical orientation. The CCK‐8 results (**Figure** **2**a) showed that compared to the pristine PMMA, the viabilities of the mBMSCs on PMMA‐2.5‐KD14, PMMA‐5‐KD14, PMMA‐2.5‐KD17, and PMMA‐5‐KD17 were improved 1.13‐, 1.19‐, 1.15‐, and 1.20‐folds, respectively. No improvement on biocompatibility was shown in PMMA‐0‐KD14 and PMMA‐0‐KD17 because of the lack of peptides. By contrast, HHC36 modified surfaces had cytotoxi‐city to mBMSCs, and compared to pristine PMMA, PMMA‐2.5‐AMP and PMMA‐5‐AMP killed 10.8% and 17.7% of the mammalian cells, limiting their application on the mono antibacterial surface (Figure S16, Supporting Information). The fluorescent images of the mBMSCs on the modified surfaces (Figure 2b; Figure S17, Supporting Information) also showed the same trend, with the adhesion of the mBMSCs improved by the fusion peptides.

**Figure 2:**
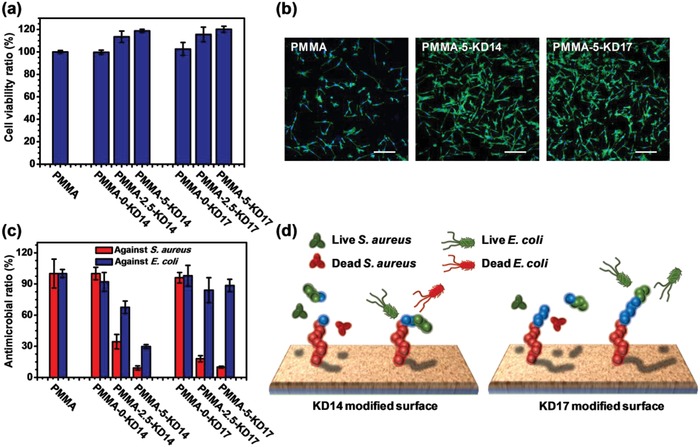
a) The CCK‐8 assay of the indicated surfaces to mBMSCs. b) The fluorescent images of mBMSCs on the indicated surfaces. Scale bar = 200 µm. c) The antibacterial activity of the indicated surfaces against *S. aureus* and *E. coli* by agar plate assay, in which the KD14‐modified surfaces exhibited broad‐spectrum antibacterial activity, and the KD17‐modified surfaces exhibited the selective antibacterial activity. d) The schematic diagram of the broad‐spectrum antibacterial activity of KD14 and the selective antibacterial activity of KD17. The data of CCK‐8 assay and antibacterial assay were expressed as mean ± SD (*n* = 3) and the experiments were repeated twice. See Sections 7 and 8 in the Supporting Information for the details of the assay.

In addition to the improved biocompatibility, we demonstrated that the KD14‐modified surfaces could recognize bacterial infection and display antibacterial activity. The in vitro antibacterial results showed that compared to pristine PMMA, PMMA‐2.5‐KD14 and PMMA‐5‐KD14 had broad‐spectrum antibacterial activity to inhibit 65.6% and 90.9% of *S. aureus*, and 32.4% and 70.4% of *E. coli*, respectively (Figure 2c), demonstrating that the recognition sequence in KD14 failed to shield the antibacterial activity of the AMP sequence (Figure 2d). A higher antibacterial activity of the KD14 against *S. aureus* was observed because some of the recognition sequence could be cleaved by the gelatinase secreting from *S. aureus* according to the HPLC and HRMS results (Figure S3, Supporting Information), suggesting that more AMP sequence was exposed to inhibit the bacteria (Figure 2d). This broad‐spectrum antibacterial activity was similar to that of the HHC36 modified surfaces PMMA‐2.5‐AMP and PMMA‐5‐AMP, which inhibited 67.9% and 89.8% of *S. aureus*, as well as 72.2% and 85.6% of *E. coli*, respectively, compared to pristine PMMA (Figure S18, Supporting Information).

Interestingly, we found that extending the number of amino acids in the recognition sequence could enhance the recognition capability of the fusion peptide. In particular, the KD17 showed dual‐recognition capability for both cell/bacteria and different bacteria. Apart from improved biocompatibility, the in vitro antibacterial results showed that compared to pristine PMMA, the KD17‐modified surfaces could kill up to 82.1% and 90.1% of the *S. aureus* on the surface (Figure 2c). According to the QCM‐D results (Table S2, Supporting Information), the KD17 exhibited more potent antibacterial activity against *S. aureus* than KD14, as PMMA‐5‐KD17 and PMMA‐5‐KD17 had similar bacterial inhibition while the density of the KD17 was lower. This might be caused by that the recognition sequence of Gly‐Pro‐Leu‐Gly‐Val being cleaved off easier than ‐Gly‐Val‐ by gelatinase (Figures S3 and S4, Supporting Information). Additionally, different from the broad‐spectrum antibacterial activity of KD14, the KD17‐modified surfaces also displayed recognition capability for different bacteria strains and exhibited negligible antibacterial activity against *E. coli* (Figure 2c). These results demonstrated that the recognition sequence in KD17 could indeed eliminate the antibacterial activity of the underlying AMP sequence. And the KD17‐modified surfaces selectively killed the *S. aureus* as the gelatinase secreted from the bacteria could cleave the recognition sequence Gly‐Pro‐Leu‐Gly‐Val (Figure 2d). To verify the role of the recognition sequence, we also synthesized the control peptide Lys‐Arg‐Trp‐Trp‐Lys‐Trp‐Trp‐Arg‐Arg‐Gly‐Gly‐Pro‐Leu‐Val‐Arg‐Gly‐Asp that contained the noncleaved sequence of Gly‐Gly‐Pro‐Leu‐Val^43^ (abbreviated as CtrlP). The HPLC and HRMS results (Figures S19 and S20, Supporting Information) showed that CtrlP had high purity, and there was no detectable change in the peptide after being treated with the gelatinase. Other than the improved biocompatibility due to the RGD sequence (Figure S21, Supporting Information), the CtrlP‐modified surfaces exhibited negligible antibacterial activities against *S. aureus* or *E. coli* (Figure S22, Supporting Information) because the Gly‐Gly‐Pro‐Leu‐Val sequence could prevent cleavage by both bacteria.

Further MD simulation also verified the shielding of the recognition sequences, and showed that the determining factor for the recognition capability to different bacteria of the KD14 and the KD17 was whether the hidden AMP sequence of the peptide was exposed. Analogous to the solvent accessible surface area (SASA),^50^ we defined the bacterial accessible surface area (BASA) to characterize the accessibility of AMP sequence. To compute BASA from MD simulations, we rolled a spherical probe with the radius of 0.14 nm to 0.6, 0.9, or 1.8 nm onto the surface of peptide conformations. The results showed that when the radius of the probe was larger than 0.6 nm, the BASA of the AMP sequence in the KD14 was larger than that in the KD17 on the 15_PMMA and 25_PMMA (**Figure** **3**a; Figure S23, Supporting Information). When the radius of the probe was 0.9 nm, the BASA of the KD14 on 15_PMMA and 25_PMMA were 9.0 and 7.9 nm^2^, respectively (Figure 3a). Also, the BASA of the KD17 on 15_PMMA and 25_PMMA were 6.1 and 5.8 nm^2^, respectively (Figure 3a). The larger BASA of the KD14 led to the loss of the recognition capability to different bacteria and the broad‐spectrum antibacterial activity. In addition, although the BASA of the KD14 was smaller than that of the KD17 on the 5_PMMA surface when the probe was larger than 0.6 nm (Figure 3a; Figure S23, Supporting Information), it was irrelevant as the peptide on the surface with low negative zeta potential exhibited poor activity (Figure 2a,c).

**Figure 3:**
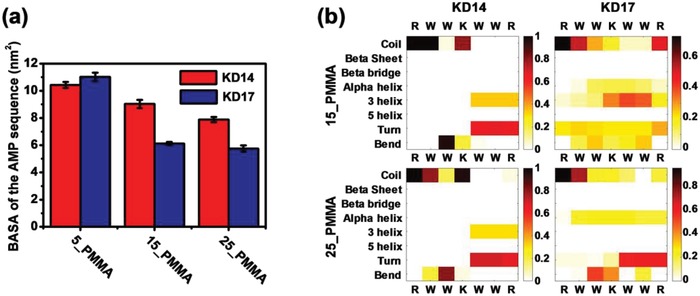
a) The bacterial accessible surface area (BASA) of the AMP sequence of KD14 and KD17 (the radius of the probe was 0.9 nm). b) The secondary structure of the AMP sequence on 5_PMMA, 15_PMMA, and 25_PMMA (the first amino acid (Lysine) and the last amino acid (Arginine) of the AMP sequence were excluded from the analysis). See Sections 9 and 10 in the Supporting Information for details of the secondary structure calculations.

Previous study reported that there was a relationship between the SASA and the secondary structure of the peptide, in which the lower SASA would lead to more helical structures.^51^ We also found that the AMP sequence in the KD14 with larger BASA contains less helical structures than that in the KD17 on 15_PMMA and 25_PMMA. According to the results (Figure 3b), the helical ratios of KD14 on 15_PMMA and 25_PMMA were 14.55% and 13.07%, respectively. And the helical ratios of KD17 on 15_PMMA and 25_PMMA were 30.54% and 19.71%, respectively. Meanwhile, analysis of g_gyrate and g_dist in Gromacs 4.5.4 showed that KD17 had a larger C—N terminal distance on the surface than KD14 (Figure S24, Supporting Information). In addition, these two peptides had similar radii of gyration.

Furthermore, we demonstrated that the versatile antibacterial PMMA substrates could inhibit the *S. aureus* infection and improve the tissue healing performance in vivo via the infective subcutaneous models on the sprague‐dawley (SD) rats. After being implanted with the bacterial solution subcutaneously for 7 days and compared to PMMA, the mono antibacterial surface (PMMA‐5‐AMP) and the versatile surfaces PMMA‐5‐KD14, PMMA‐2.5‐KD17, and PMMA‐5‐KD17 could kill 96.9%, 95.0%, 92.5%, and 96.2% of *S. aureus*, respectively (**Figure** **4**a; Figure S25, Supporting Information). PMMA‐0‐KD17 with the lack of peptides displayed negligible antibacterial activity. Meanwhile, the tissue healing performance was visually observed by the surgical cut‐opening of the implanted site, as obvious tissue healing was observed in the implanted site of the antibacterial surfaces (PMMA‐5‐AMP, PMMA‐5‐KD14, PMMA‐2.5‐KD17, and PMMA‐5‐KD17) compared to the severity of infection and tissue necrosis in PMMA and PMMA‐0‐KD17 groups (Figure 4b). The pathological results of encapsulated tissues at each interval obtained from the hematoxylin and eosin (H&E) staining (Figure 4c) showed that in addition to serious bacterial infection, there was obvious inflammatory cells infiltration (the blue arrow) in PMMA or PMMA‐0‐KD17 groups, and the musculature was destroyed. Although PMMA‐5‐AMP exhibited antibacterial activity and there was no obvious inflammation (Figure 4c), the endothelial dysfunction and a large amount of cutaneous petechia (the red arrow) were found, which were consistent with the in vitro results showing that the HHC36 modified surface exhibited evident cytotoxicity. These results also illustrated the limitation of mono antibacterial biomaterials. By contrast, the versatile surfaces could heal the tissues, and the H&E staining results showed that there were no evident inflammatory cells and endothelial dysfunction, and evident fibrosis/fibrous capsules were developed (Figure 4c). It was well known that HHC36 could inhibit bacterial infection, and RGD could promote macrophage recruitment to reduce the incidence of the inflammation of the biointerface. In the present study, we illustrated that these two sequences could have the synergistic effect in vivo by fusing the two peptides.

**Figure 4:**
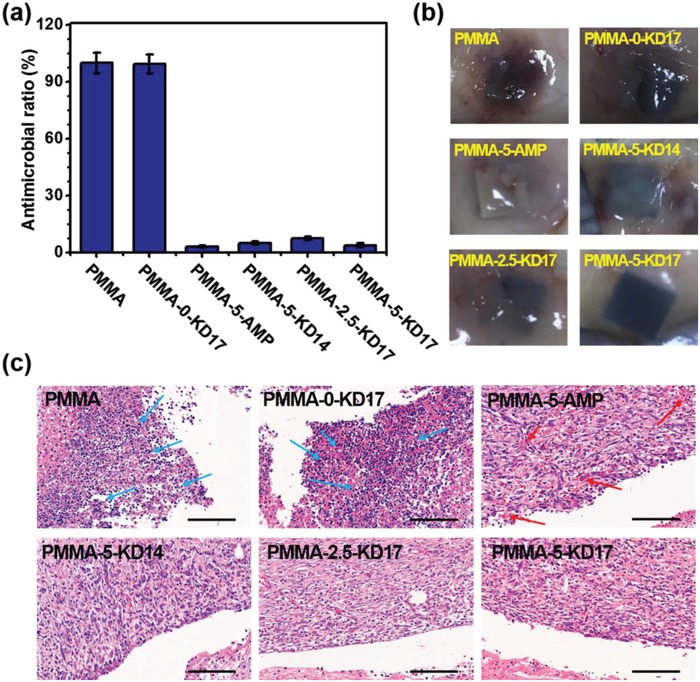
a) The surviving *S. aureus* on the indicated surfaces via the subcutaneous implantation in the mice for 7 days (*n* = 3). b) The images of the indicated surfaces subcutaneously implanted after 7 days. c) The microscopy images of the H&E staining for the indicated surfaces implanted after 7 days (the blue arrows in the groups of PMMA and PMMA‐0‐KD17 denoted the inflammatory cells, and the red arrows in the group of PMMA‐5‐AMP denoted the cutaneous petechia). See Section 11 in the Supporting Information for details of the in vivo assay.

In summary, the smart versatile surfaces we designed could be prepared conveniently by 2D self‐assembly with the ability to recognize cells/bacteria, while displaying improved biocompatibility and antibacterial activity. Interestingly, by increasing the number of the amino acids in the recognition sequence, the fusion peptides‐modified surfaces could also recognize different bacterial strains to exhibit selective instead of the broad‐spectrum antibacterial activity. We also revealed by MD simulation that it was due to the difference in the BASA of the AMP sequence in the peptide. Meanwhile, the versatile surfaces we prepared showed excellent antibacterial activity and biocompatibility in vivo to improve tissue healing. Our work establishes a novel and convenient strategy to prepare the smart versatile materials through a well‐defined mechanism.

## Experimental Section

To prepare the versatile surface in the experiment, 50 µL of the PMMA solution in methylbenzene (1 wt%) was dropped onto the titanium surface and was spun at a speed of 4000 rpm for 30 s. After cleaning and drying, the surfaces were treated by oxygen plasma for 0, 2.5, and 5 min, and immersed into 100 µL of the AMP, CtrlP, KD14, or KD17 solution with the concentration of 500 × 10^−6^ m in water for 30 min. To compute the conformation of the peptide on the surface, all‐atom MD simulations of the binding of both KD17 and KD14 were performed on the surfaces with different charge densities: 5, 15, or 25 negative charges on the area of 64 nm^2^. To obtain binding free energy, MM/PBSA calculations were performed based on conformations from MD simulations. For each system, 4100 ns MD simulations were performed. All SD rat's experiments were approved by Guangdong Medical Laboratory Animal Center in Foshan. The details of the experiments and simulations were shown in the Supporting Information.

## Conflict of Interest

The authors declare no conflict of interest.

## Supporting information

Supplementary
