# Supplementary material for: Mechanistic Insights and Rational Design of a Versatile Surface with Cells/Bacteria Recognition Capability via Orientated Fusion Peptides
Source: Adv Sci (Weinh). 2019 Mar 4;6(9):1801827. doi: 10.1002/advs.201801827 (PMC6498104; doi:10.1002/advs.201801827)
Supplement: Supplementary file 1 — Supplementary [file ADVS-6-1801827-s001.pdf]

Copyright WILEY-VCH Verlag GmbH & Co. KGaA, 69469 Weinheim, Germany, 2019.

ADVANCED SCIENCE
Open Access

Supporting Information

for *Adv. Sci.,* DOI: 10.1002/advs.201801827

Mechanistic Insights and Rational Design of a Versatile Surface with Cells/Bacteria Recognition Capability via Orientated Fusion Peptides

Lin Wang, Junjian Chen, Xiangze Zeng, Peter Pak-Hang Cheung, Xiaoyan Zheng, Liangxu Xie, Xuetao Shi, Li Ren, Xuhui Huang,* and Yingjun Wang**

Supporting Information

Mechanistic Insights and Rational Design of the Versatile Surface with Cells/Bacteria Recognition Capability via Orientated Fusion Peptides

Lin Wang,†[a,b] Junjian Chen,†[c] Xiangze Zeng,[b] Peter Pak-Hang Cheung,[b] Xiaoyan Zheng,[b] Liangxu Xie,[b] Xuetao Shi,[a] Li Ren,*[c] Xuhui Huang*[b] and Yingjun Wang*[a]

1. Materials

The peptides were synthesized from ChinaPeptides Co., Ltd. (Shanghai, China). Acetone, ethanol and methylbenzene were purchased from Guangzhou Chemical Factory Co. Ltd. (Guangdong, China). High-glucose Dulbecco's modified Eagle's medium (H-DMEM), fetal bovine serum (FBS) and Trypsin-EDTA were purchased from Gibco® by Life Technologies (New York, USA). Nutrient broth and nutrient agar for bacterial culture were purchased from Huankai Microbial Sci. & Tech. Co. Ltd. (Guangdong, China). The CCK-8 kit was purchased from Dojindo (Shanghai, China). The *mouse bone mesenchymal stem cells* (*mBMSCs*, strain ATCC CRL-12424), *Staphylococcus aureus* (*S. aureus*, strain ATCC 29213) and *Escherichia coli* (*E. coli*, strain ATCC 15224) were purchased from VWR International, LLC (Padnor, PA, USA).

2. The high-performance liquid chromatography (HPLC) and the high resolution mass spectrometry (HRMS) assay of the peptides

The following solutions were prepared: (0.493 mg/mL for KD14, 0.560 mg/mL for KD17, 0.560 mg/mL for CtrlP, 0.25 mM) peptide. The following mixture was prepared: (0.493 mg/mL for KD14, 0.560 mg/mL for KD17, 0.560 mg/mL for CtrlP, 0.25 mM) peptide, (1 mg/mL) gelatinase in buffer (50 mM TES, 0.36 mM Calcium chloride, pH 7.4) with a total volume of 0.5 mL. After 1 h to reaction of the mixture, the origin peptide and peptide sequences was analyzed by HPLC (acetonitrile (0.1% TFA) /water (0.1% TFA) linear gradient from 23%/77% to 48%/52% in 25 min flux 1 mL/min, 280 nm detection). For peptide of KD14, only one peak at t = 7.6 min were observed before treated by gelatinase; after treated by gelatinase, there were two peaks at t = 7.6 min and t = 8.4 min, responsively corresponding to HRMS of $[M + H]^{2+}$ = 987.0308 (the exact mass of KRWWKWWRRGVRGD (KD14) = 1972.2823) and $[M + H]^{2+}$ = 772.9329 (the exact mass of KRWWKWWRRG = 1544.8263). For peptide of KD17, before treated by gelatinase, only one peak at t = 9.6 min were observed with the HRMS of $[M + H]^{2+}$ = 1120.6201 (the exact mass of KRWWKWWRRGPLGVRGD (KD17) = 2239.6038); after treated by gelatinase, there was one peak at t = 10.7 min corresponding to HRMS of $[M + H]^{2+}$ = 907.0097 (the exact mass of KRWWKWWRRGPLG = 1812.1482). For peptide of CtrlP, before and after treated by gelatinase, the same peak at t = 5.4 min were observed with the HRMS of $[M + H]^{3+}$ = 747.4088 and 747.4183 (the exact mass of KRWWKWWRRGGPLVRGD (CtrlP) = 2239.6038).

3. Preparations of substrate

The titanium wafers (5 mm × 5 mm × 1 mm) were purchased from Zhongjingkeyi Technology Co., Ltd. (Beijing, China). The polymethyl methacrylate (PMMA) surface was prepared by spinning PMMA (Beiaolu, Shanghai, $M_w = 8 \times 10^4$) solution onto the titanium wafer. Briefly, 50 μL of PMMA solution in methylbenzene (1 wt%) was dropped onto the titanium wafer and was spun at a speed of 4000 rpm for 30 s.

For the QCM-D assay, the PMMA electrodes were prepared by spinning as above on Ti electrodes, which were purchased from Biolin Scientific (Goteborg, Sweden).

4. Preparation of the surface

The PMMA surfaces were washed with distilled water following preparation and were dried by nitrogen. After cleaning and drying, the surfaces were treated by oxygen plasma for 0, 2.5 or 5 min, which was abbreviated as PMMA-0min (pristine PMMA), PMMA-2.5min and PMMA-5min, respectively. Then, the surfaces were immersed into 100 μL of the HHC36, CtrlP, KD14 or KD17 solution at a concentration of 500 μM in water for 30 min for the self-assembly. Afterwards, the surfaces were washed with distilled water for 3 times and dried by nitrogen.

5. Quartz crystal microbalance with dissipation (QCM-D) assay

The QCM electrodes were treated by oxygen plasma for 0, 2.5 and 5 min, and placed into sensor chambers of the Q-Sense E4 QCM-D system (Q-Sense AB, Sweden). Distilled water was introduced to the electrode until the frequency was balanced. After that, 100 μM of different peptides (KD14 or KD17) solution in water was introduced onto the electrode at a speed of 30 μL/min at 25 °C. After being balanced, the electrodes were rinsed again with distilled water to reach balance again.

6. Characterization of the surfaces

The water contact angles of the surfaces were recorded on the OCA15 contact angle goniometer (Dataphysics, Filderstadt, Germany) at room temperature with 1 μL of distilled water as the reference liquid. Atomic Force Microscope (AFM) images were obtained by a MutiMode Nanoscope IIIa AFM (Digital Instruments Inc., Santa Barbara, CA). Zeta potentials

of different peptides and surfaces were measured on a SZetasizer Nano ZS (Malvern Instruments Inc., Malvern, UK).

7. *In vitro* cell assay

Mouse bone mesenchymal stem cells (*mBMSCs*) were cultured in H-DMEM medium with 10% FBS under 5% CO_2 at 37 °C. The medium was replaced every three days. Cells were passaged after the coverage reached 80% confluence and 3-5 passaged cells were used for the experiments.

All the samples used for the cell assay were sterilized with 75% ethanol for 2 h. After sterilization, the samples were removed to a new 24-well plate and washed twice with PBS. Then, the *mBMSCs* were added directly onto each surface (10000 cells per well for the CCK-8 assay and 2×10^4 cells for the confocal microscopy assay) and cultured under 5% CO_2 at 37 °C.

The biocompatibility of the sample was evaluated using a CCK-8 kit. Briefly, after the culturing of 24 h, samples were transferred to a new 24-well plate and washed three times with PBS, and then they were immersed in 400 μL of complete medium containing 40 μL of CCK-8 solution. After 3 h incubation at 37 °C, 100 μL of the solution was transferred to a 96-well plate and the optical density (OD) was measured with an ELISA plate reader (Varioskan Flash 3001, Thermo, Finland) at 450 nm.

The cells on the samples were also characterized by the confocal microscopy assay. Briefly, after the culturing of 24 h, the cells/substrates were transferred to the new 24-well plates, washed 3 times with PBS and fixed with 4% formaldehyde for 1 h. After being washed with PBS for 3 times, the substrates were immersed first into 0.1% Triton X-100 for 10 min to increase the permeability of cells' membrane, then in a phalloidin-FITC probe for 1 h and finally in DAPI for 10 min. The fluorescent images were obtained by laser scanning confocal microscopy (Leica TCS SP5, Germany).

8. *In vitro* antibacterial assay

S. aureus and *E. coli* were utilized as target bacteria. A single colony of bacteria was used to inoculate in 5 mL of nutrient broth at 37 °C overnight with shaking (150 rpm). Following growth,

0.1 mL of bacterial suspension was inoculated into 15 mL of nutrient broth and incubated for 5 h with shaking (250 rpm) at 37 °C to achieve mid-log phase growth.

The antibacterial activity of KD14, KD17 or HHC36 in solution was tested. Briefly, different peptide was mixed with bacteria at different concentrations (0 μM, 5 μM, 10 μM, 15 μM and 50 μM) in the nutrient broth, and the concentration of bacteria was 1 × 10^6 CFU/mL. After cultured at 37 °C for 2.5 h, the antibacterial activity was determined by diluting the bacterial suspension to 10^0, 10^1, 10^2, 10^3 and 10^4 times with PBS, and 10 μL of the bacterial suspension was taken to evaluate the viability of bacteria by agar plates.

Before performing the antibacterial assay, the samples were placed in the 48-well plates and washed with 75% ethanol for three times. Then, 10 μL of the bacterial suspension (1 × 10^6 CFU/mL in the nutrient broth) was added onto each sample to cover the surface. To avoid the evaporation of the bacterial suspension, the wells and the gaps around the well containing the sample were filled with PBS solution. After the culturing of 2.5 h at 37 °C, the samples and bacterial suspension were moved to a new tube with the bacteria. Then, the system was treated by sonication for 3 min and followed by vortexing for 1 min to detach the adhered bacteria. The detached bacteria were collected and diluted to the desired concentration. 10 μL of the bacterial suspension was collected to evaluate the viability of bacteria using agar plates. After incubation at 37 °C for 15 h, the number of bacteria on each agar plate was counted.

9. All-atom molecular dynamics (MD) simulation

All the MD simulations were performed by the Gromacs 4.5.4 package[1] with the general Amber force field.[2] The partial charge was derived by RESP fitting[3] to the electrostatic potential computed by HF/6-31G*. The initial structures of the syndiotactic PMMA molecule with 10 units and the peptide were optimized at a HF/6-31G* level by the Gaussian package.[4] Then, the substrate composed of 135 PMMA molecules with the size of 8 × 8 × 3 nm^3 and density of 1.18 g/cm^3 was built by the “genbox” program in Gromacs. This substrate was energy minimized by the steepest descent method followed by a 200-ns NVT simulation. Next 5, 15 or 25 negative charges were added on the surface of the substrate by demethylation of the methyl ester group in the PMMA molecule randomly, and the substrates were abbreviated as 5_PMMA, 15_PMMA and 25_PMMA, respectively. The partial charge of the PMMA molecule after demethylation was re-derived by RESP fitting[3] to a HF/6-31G* electrostatic potential.

To obtain initial conformations of KD14 and KD17, we first performed MD simulations of these two peptides in bulk solution. In particular, we employed Modeller[5,6] to prepare a helical conformation for both peptides. We then dissolved each of them in a simulation box with the dimension of 5 × 5 × 5 nm^3 and containing 3557 and 4675 TIP3P water molecules[7] for KD14 and KD17 respectively. For both systems, 5 Cl^- ions were added to neutralize the system. We performed the energy minimization by the steepest descent method followed by a 200-ps NPT simulation with the positions of all heavy atoms restrained. We then performed a 30-ns NPT simulation. In these NPT simulations, the temperature was maintained at 300 K by the V-rescale thermostat,[8] while the pressure was set at 1 bar using the Parrinello-Rahman method.[9] The LINCS algorithm[10] was used to restrain all the bonds, and the PME method[11] was applied to calculate the long-range electrostatic interactions. The cutoffs of the short-range electrostatic and van der Waals interactions were set to 1.2 nm and 1.1 nm, respectively. We choose the last conformation of this simulation as the initial conformation for further modeling of the binding of these two peptides to the surface as described below.

To model the binding of these two peptides to the surface, we first placed them above the surface by randomly choosing an orientation (the distance between the center of mass of the peptide and the surface was 2 to 3 nm) with the conformation discussed above. We then dissolved each of the peptides in a simulation box of 8 × 8 × 10 nm^3 containing TIP3P water molecules.[7] For KD14, the system contains 14665, 14666, and 14665 water molecules for the 5_PMMA, 15_PMMA and 25_PMMA surface, respectively. For the KD17, the system contains 14658, 14659, and 14658 water molecules for the 5_PMMA, 15_PMMA and 25_PMMA surface, respectively. For all the systems, we added Cl^- ions make the simulation box neutral. We then performed energy minimization using the steepest descent method followed by a 500-ps position restrained NVT simulation, and then another 1-ns position restrained NPT simulation. For each system, we performed four 100-ns production MD simulations in the NVT ensemble with different initial orientations of the peptide relative to the surface with an aggregated simulation time of 2,400 ns. The other set-up of MD simulations were identical as those discussed in the previous paragraph.

10. Analysis of the MD simulation trajectories

Each peptide were observed to adsorb on the PMMA surface in the 100ns MD simulations, which can be monitored by the distance of the center of mass (COM) of peptide and COM of

PMMA along the Z-axis. The coordinates at the Z-axis between the COM of two peptide (KD14 and KD17) and the COM of the three types of substrates (5_PMMA, 15_PMMA and 25_PMMA) were calculated by "g_traj" tool that implemented in Gromacs 4.5.4, respectively. [1] The mean and the standard deviation of the distance was calculated from the 4 independent MD trajectories at indicated time points.

The contact area between the peptide and the substrate was the difference of the solvent accessible surface area between the peptide without PMMA surface and the peptide adsorbed on the PMMA surface, which was calculated by g_sas program implemented in Gromacs 4.5.4.[1] The mean and the standard deviation of the contact surface area was calculated from the 4 independent MD trajectories at indicated time points.

The secondary structures of the KD14 and KD17 were calculated by DSSP program.[12] The radius of gyration of the peptide was calculated by the "g_gyrate" program implemented in Gromacs 4.5.4.[1] For each of the four MD simulations of each peptide adsorbing on a particular surface, we extracted 4,000 conformations from its last 80 ns segment to compute the secondary structure and radius of gyration.

The binding free energy between the peptide and the surface was calculated by the MM/PBSA method with the "g_mmpbsa" program[13] implemented in Gromacs 4.5.4. In order to properly estimate long-range electrostatic interactions between two groups (i.e. AMP sequence in the peptide and the substrate) in PME, we computed electrostatic interactions for three systems: one containing both AMP sequence and substrate (E_{elec_total}), one containing only AMP sequence (E_{elec_AMP}), and one containing only the substrate (E_{elec_sub}). We can then compute the electrostatic interactions between AMP sequence and substrate by: $E_{elec\_total} - E_{elec\_AMP} - E_{elec\_sub}$. The dielectric constant was set as 1 (ε=1). [14, 15] For each peptide adsorbing on a particular substrate, the MM/PBSA calculations were performed on a total of 320 conformations extracted from the last 80 ns of four MD simulations. By using the same method, we obtained the binding free energy between the RGD sequence in the peptide and the substrate. We note that it has been suggested recently that choosing a larger value of dielectric constant (e.g. ε=2) may yield more consistent results with experiment especially for systems with extended binding interface. [16] Therefore, we also performed our calcualtions with ε=2. As shown in **Figure S14** and **Figure S15**, the binding free energies between the AMP sequence in KD14 and 5_PMMA, 15_PMMA, 25_PMMA are -279.5 kJ/mol, -680.0 kJ/mol, -939.5 kJ/mol, respectively. Meanwhile, the binding free energies between KD17 and

5_PMMA, 15_PMMA, 25_PMMA are -189.0 kJ/mol, -639.9 kJ/mol, -1029.0 kJ/mol, respectively. The conclusions drawn from ε=2 remains the same trend as that from ε=1.

To compute the bacterial accessible surface area (BASA) of the AMP sequence, we adopted a similar approach for computing the solvent accessible surface area[17] by rolling a probe sphere with the radius of 0.14 nm, 0.6 nm, 0.9 nm or 1.8 nm over the surface of the peptide. To achieve this, we applied the “g_sas” program implemented in Gromacs 4.5.4 on a total of 1,600 conformations extracted from the last 80 ns of four MD simulation for each system, where the peptide was found to be bound to the surface.

11. *In vivo a*ssay

All SD rat's experiments were approved by Guangdong Medical Laboratory Animal Center in Foshan. According to the Agreement of Ethical and Moral Obligation, all animals should be euthanized after the experiment was complete and all operations must strictly obey the rules in order to avoid secondary damage. Animals were maintained in Guangdong Medical Laboratory Animal Center in Foshan. All procedures involving experimental animals were performed under strict sterile conditions in a conventional operation theater. We anaesthetized SD rats (female, 180-220 g) with 10% chloral hydrate in a dosage of 0.1 mL/kg. After that, we shaved the hair at the back of the rats, and disinfected with povidone iodine solution prior to the operation. Then a 1 cm long full depth incision was made on the back, 10 μL of *S. aureus* solution (1×10^8 CFU/mL) was injected into the incision, and the sample was implanted subcutaneously into the incision. Finally, the incision was closed with silk 3-0 suture.

After surgery, the SD rats were cultured in cages in a temperature-controllable facility with access to antibiotic-free food and water. After 7 days of the implantation, the SD rats were euthanized and the sample was extracted from the incision site. The sample was immersed in LB media and cultured for 6 h. Then the LB media was collected, diluted to 10^1, 10^2, and 10^3 times with PBS, and 10 μL of each solution was taken to evaluate the viability of bacteria with blood agar plates.

We preformed pathological examinations to evaluate the *in vivo* infection with the inoculated *S. aureus*. The incision part of each group was immersed in 4% formaldehyde 2 days, placed into processing cassettes, dehydrated by a serious of ethanol gradients (50% for 2 h, 70% for 2 h, 80% for 1.5 h, 95% for 1 h twice, and 100% for 45 min twice), placed in dimethylbenzene three times for each 30 min, and embedded in paraffin wax boxes for 2 h at 60 °C. Then, the

embedding process was implemented as follows: the temperature was adjusted to 63 °C to melt the paraffin wax, the tissue was immersed in the melting paraffin wax, and then transferred to a freezing platform to cool and solidify. Once solid, 4 μm tissue sections (slices) were acquired by microtome, and the slices were baked at 60 °C for 1 h.

H&E staining was employed to evaluate the histological morphology of *in vivo* infection. The slices were subsequently immersed in dimethylbenzene I for 15 min (50 °C), dimethylbenzene II for 5 min, dimethylbenzene III for 5 min, 100% ethanol for 5 min, 95% ethanol for 5 min, 80% ethanol for 5 min, 70% ethanol for 5 min, and distilled water for 5 min. After that, the slices were immersed in hematoxylin for 5 min, rinsed with distilled water for 1 min, stained with eosin solution for 5 min, and rinsed with distilled water for 1 min. Finally, the slices were dehydrated by 80% ethanol for 5 min, 95% ethanol for 5 min, 100% ethanol for 5 min twice, dimethylbenzene I for 10 min, dimethylbenzene II for 10 min.

References:

[1] S. Pronk, S. Pall, R. Schulz, P. Larsson, P. Bjelkmar, R. Apostolov, M. R. Shirts, J. C. Smith, P. M. Kasson, D. van der Spoel, B. Hess, E. Lindahl, *Bioinformatics* **2013**, *29*, 845.

[2] J. M. Wang, R. M. Wolf, J. W. Caldwell, P. A. Kollman, D. A. Case, *J. Comput. Chem.* **2004**, *25*, 1157.

[3] C. I. Bayly, P. Cieplak, W. D. Cornell, P. A. Kollman, *J. Phys. Chem.* **1993**, *97*, 10269.

[4] M. J. Frisch, G. W. Trucks, H. B. Schlegel, G. E. Scuseria, M. A. Robb, J. R. Cheeseman, et al, Gaussian 09, Revision A.02. Gaussian Inc., Wallingford CT, **2004**, *26*.

[5] B. Webb, A. Sali, *Current Protocols in Bioinformatics, John Wiley & Sons, Inc.* **2014**, 5.6.1.

[6] M. A. Marti-Renom, A. Stuart, A. Fiser, R. Sánchez, F. Melo, A. Sali, *Annu. Rev. Biophys. Biomol. Struct.* **2000**, *29*, 291.

[7] W. L. Jorgensen, J. Chandrasekhar, J. D. Madura, R. W. Impey, M. L. Klein, *J. Chem. Phys.* **1983**, *79*, 926.

[8] G. Bussi, D. Donadio, M. Parrinello, *J. Chem. Phys.* **2007**, *126*, 014101.

[9] M. Parrinello, A. Rahman, *Phys. Rev. Lett.* **1980**, *45*, 1196.

[10] B. Hess, H. Bekker, H. J. C. Berendsen, J. Fraaije, *J. Comput. Chem*. **1997**, *18*, 1463.

[11] T. Darden, D. York, L. Pedersen, *J. Chem. Phys.* **1993**, *98*, 10089.

[12] W. Kabsch, C. Sander, *Biopolymers* **1983**, *22*, 2577.

[13] R. Kumari, R. Kumar, A. Lynn, *J. Chem. Inf. Model.* **2014**, *54*, 1951.

[14] P. A. Kollman, I. Massova, C. Reyes, B. Kuhn, S.H. Huo, L. Chong, et al. *Acc. Chem. Res.* **2000**, 33, 889.

[15] J. Srinivasan, T. E. Cheatham, P. Cieplak, P. A. Kollman, D. A. Case. *J. Am. Chem. Soc.* **1998**, 120, 9401.

[16] S. Genheden, U. Ryde. *Expert Opin. Drug Discov.* **2015**, 10, 449-461.

[17] T. J. Richmond, *J. Mol. Biol.* **1984**, *178*, 63.

[18] J. Felsenstein, *Evolution* **1985**, *39*, 783.

Figure S1 (a)The high-performance liquid chromatography and (b) the high resolution mass spectrometry results of KD14. See Sec.2 for details of the assay.

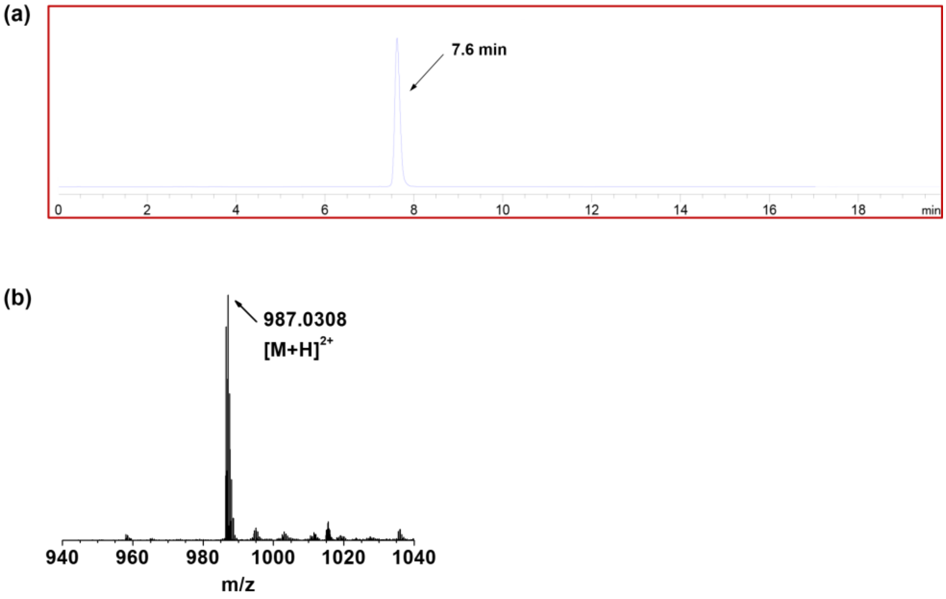

Figure S2 (a)The high-performance liquid chromatography and (b) the high resolution mass spectrometry results of KD17. See Sec.2 for details of the assay.

(a)

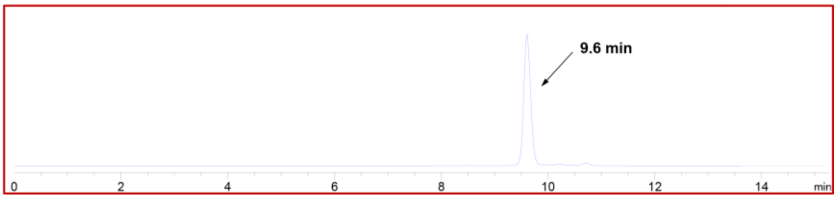

(b)

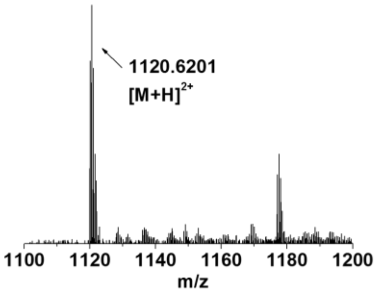

Figure S3 (a)The high-performance liquid chromatography and (b) the high resolution mass spectrometry results of KD14 incubated with gelatinase for 1 h. See Sec.2 for details of the assay.

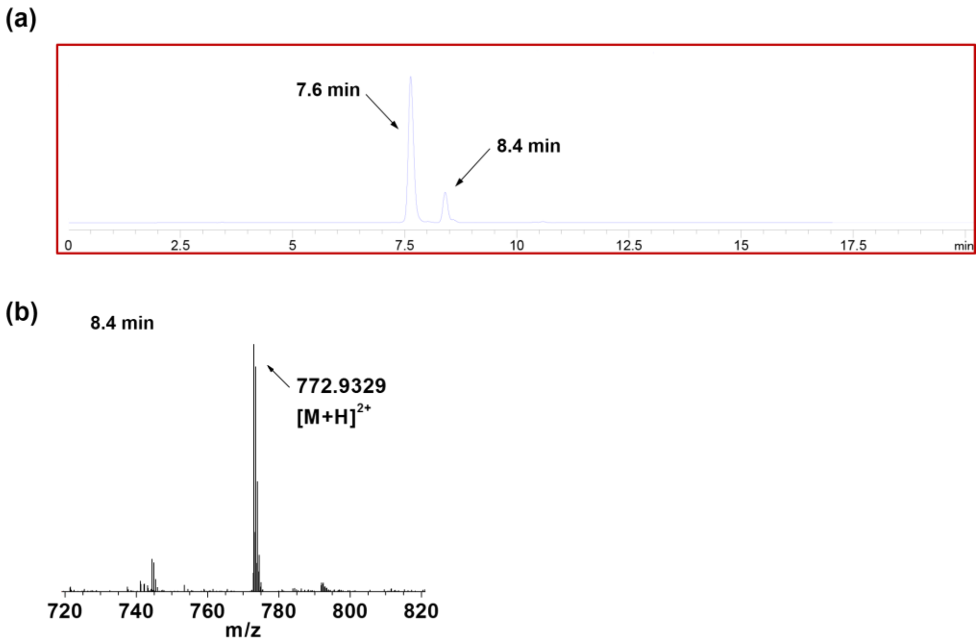

Figure S4 (a)The high-performance liquid chromatography and (b) the high resolution mass spectrometry results of KD17 incubated with gelatinase for 1 h. See Sec.2 for details of the assay.

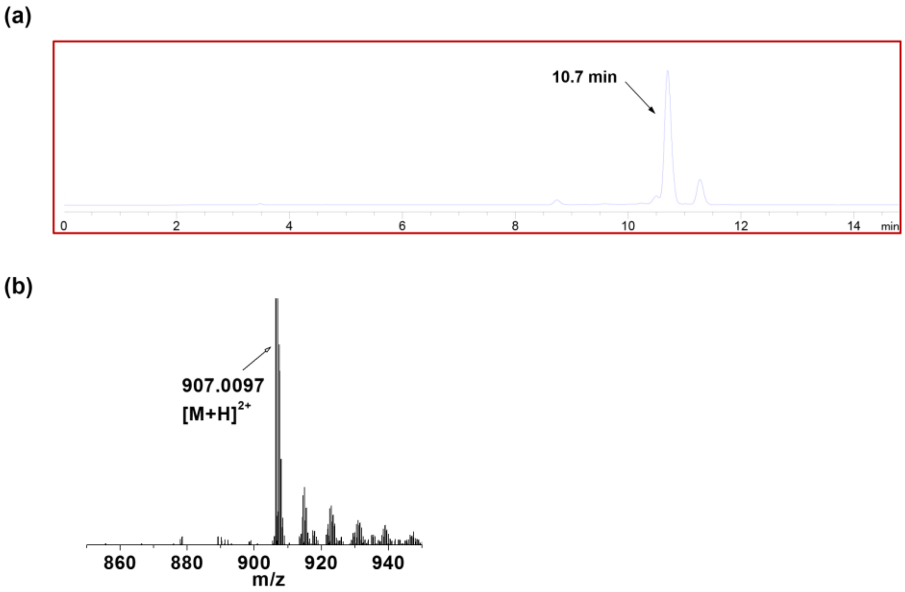

Figure S5 The antibacterial activity of (a) HHC36 peptide, (b) KD14 and (c) KD17 in solution against *S. aureus* and *E. coli* by the agar plate assay. See Sec.8 for details of the antibacterial assay.

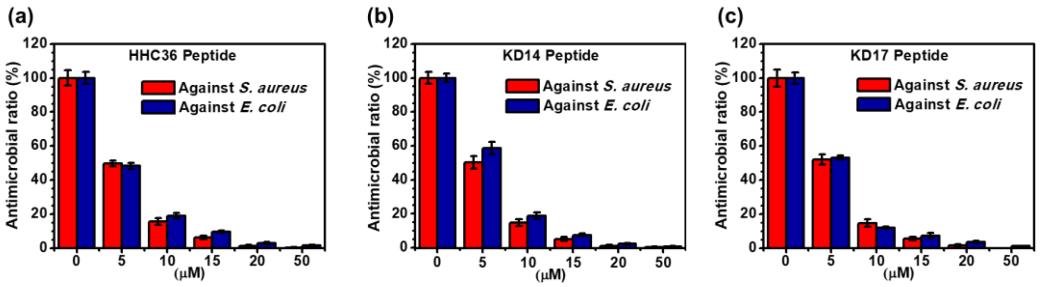

Figure S6 The zeta potential of the PMMA surface after being treated with Oxygen plasma for 0 min (PMMA), 2.5 min (PMMA-2.5min) or 5 min (PMMA-5min). See Sec.6 for details of the zeta potential assay.

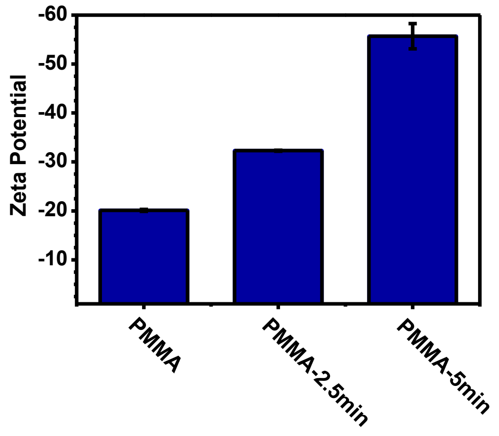

Figure S7 The water contact angles of the PMMA, PMMA-5min, PMMA-5-KD14 and PMMA-5-KD17 at room temperature with distilled water. (* denotes $p < 0.05$ and & denotes $p < 0.001$). See Sec.6 for details of the contact angle assay

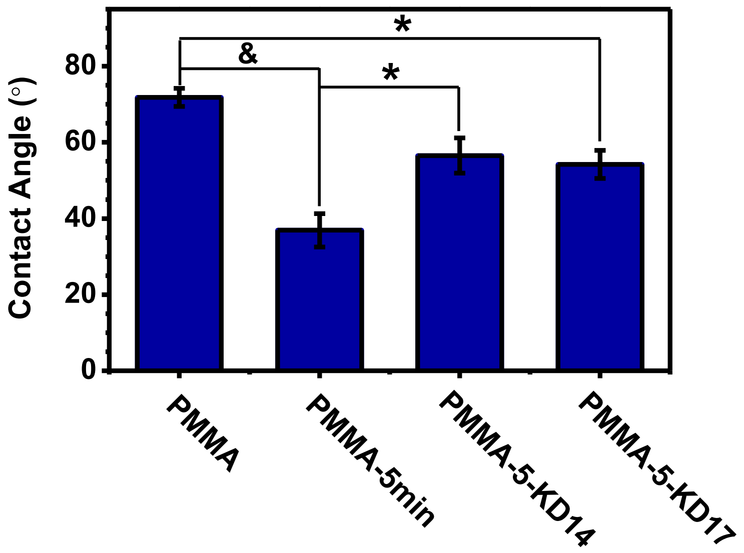

Figure S8 (a) The structure of the syndiotactic PMMA molecule with 10 units for the substrate in simulation, and the side cut view of the substrate; (b) the top view of the substrate. One single chain of PMMA on the surface was highlighted in cyan color with bond and sphere shape. VMD was used for the visualization. See Sec. 9 for the details of PMMA molecules and substrate.

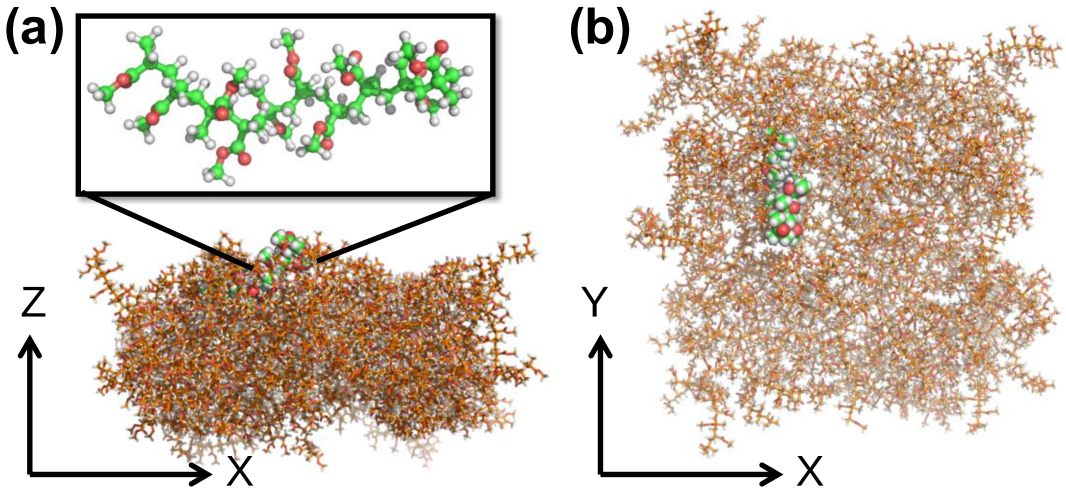

Figure S9 The representative snapshots at indicated time points in MD trajectories: (a) KD14 on 5_PMMA; (b) KD14 on 15_PMMA; (c) KD14 on 25_PMMA; (d) KD17 on 5_PMMA; (e) KD17 on 15_PMMA and (f) KD17 on 25_PMMA. The AMP sequence was marked by red, the recognition sequence was marked by blue, and the RGD sequence was marked by green. See Sec. 9 for the details of the MD simulations.

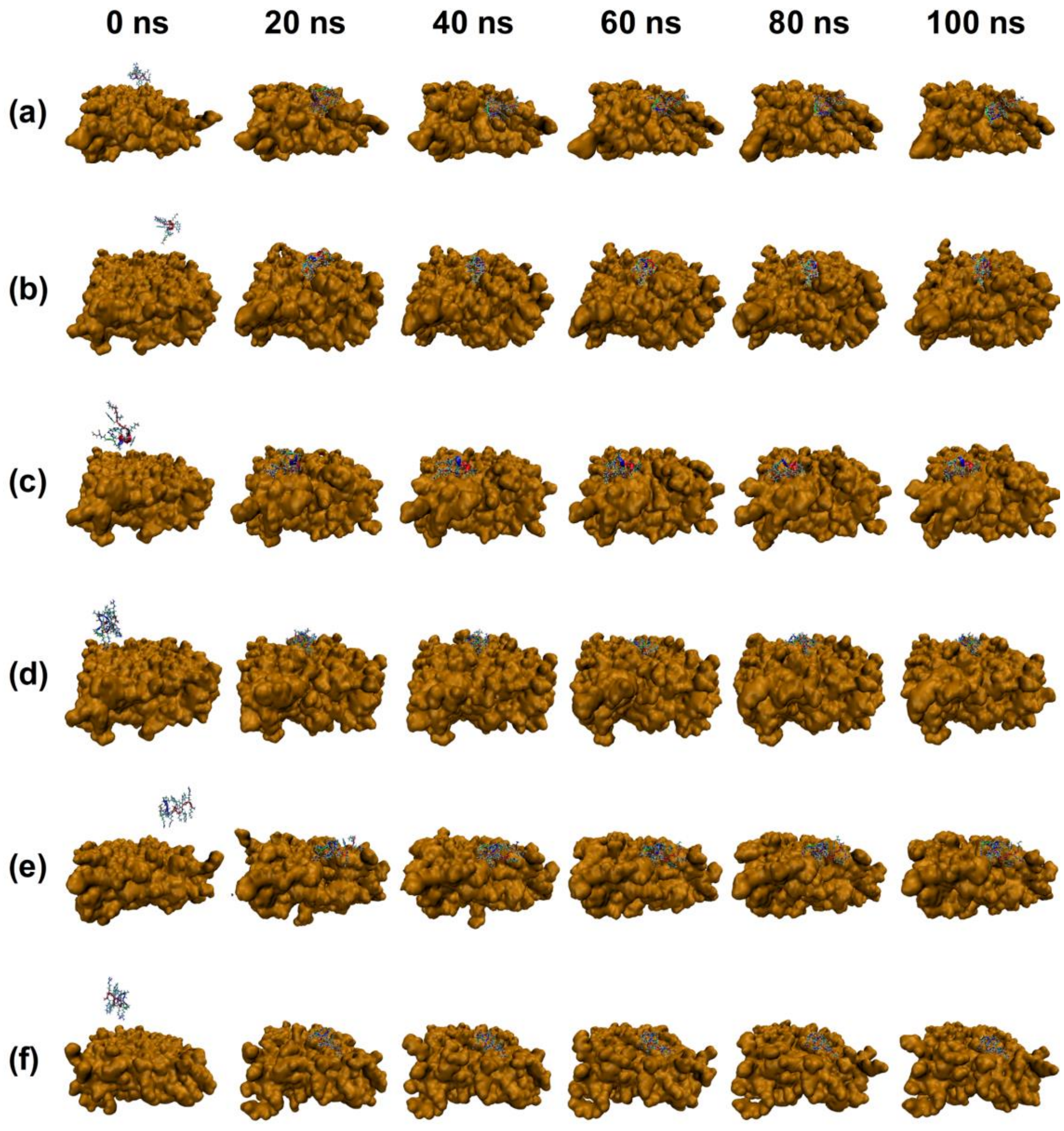

Figure S10 The evolutions of the distance between the center of mass (COM) of peptide and the COM of PMMA substrate along Z axis from trajectories. The value of the distance is obtained by averaging the four trajectories for each of the system. See Sec.9 and Sec.10 for details of the COM calculations.

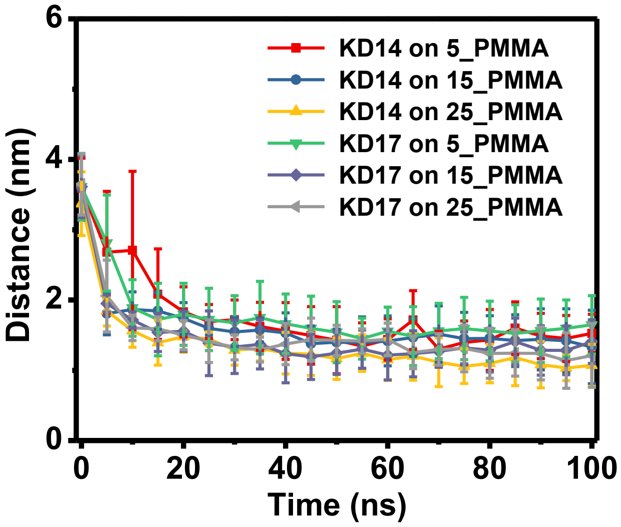

Figure S11 The evolutions of the contact area between peptide and PMMA substrate from trajectories in 100 ns. The value of the contact area is obtained by averaging the four trajectories for each of the system. See Sec.9 and Sec.10 for details of the contact area calculations.

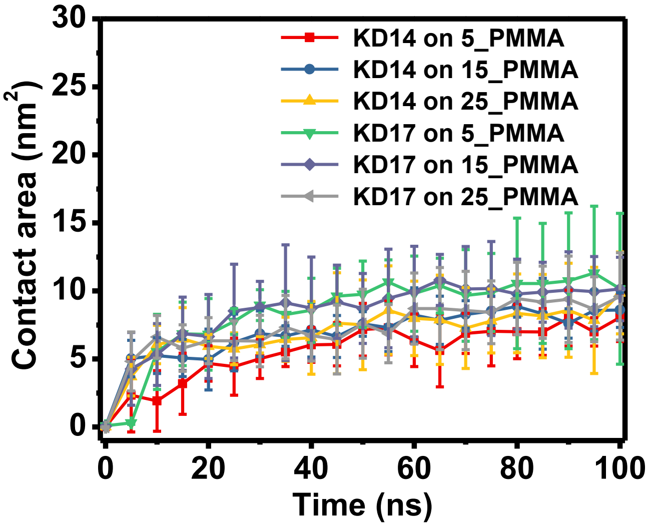

Figure S12 The binding free energy obtained from MM/PBSA method: (a) the binding free energy between the indicated surface and the AMP sequence in KD14; (b) the binding free energy between the indicated surface and the RGD sequence in KD14; (c) Contributions of electrostatic interactions to binding free energy between the indicated surface and the AMP sequence in KD14. The dielectric constant was 1 (ε=1). The error bars were estimated by the bootstrapping method[18]. Specifically, we bootstrapped 20 times, and at each time 40 MD conformations were randomly chosen. See Sec.9 and Sec.10 for details of the MM/PBSA calculations.

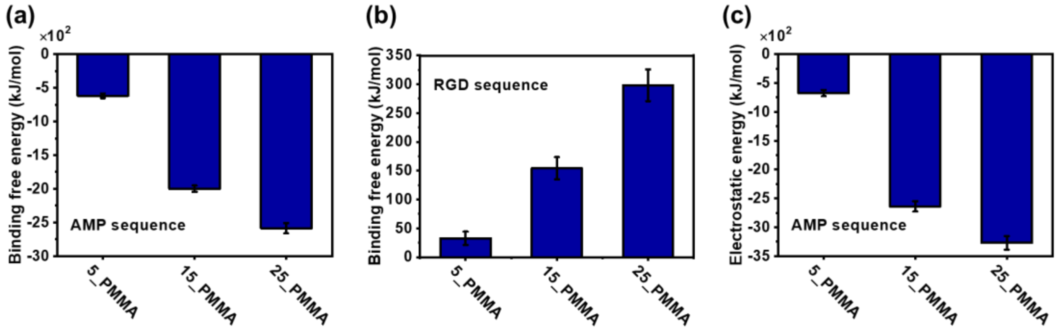

Figure S13 The binding free energy obtained from MM/PBSA method: (a) the binding free energy between the indicated surface and the AMP sequence in KD17; (b) the binding free energy between the indicated surface and the RGD sequence in KD17; (c) Contributions of electrostatic interactions to binding free energy between the indicated surface and the AMP sequence in KD17. The dielectric constant was 1 (ε=1). The error bars were estimated by the bootstrapping method[18]. Specifically, we bootstrapped 20 times, and at each time 40 MD conformations were randomly chosen. See Sec.9 and Sec.10 for details of the MM/PBSA calculations.

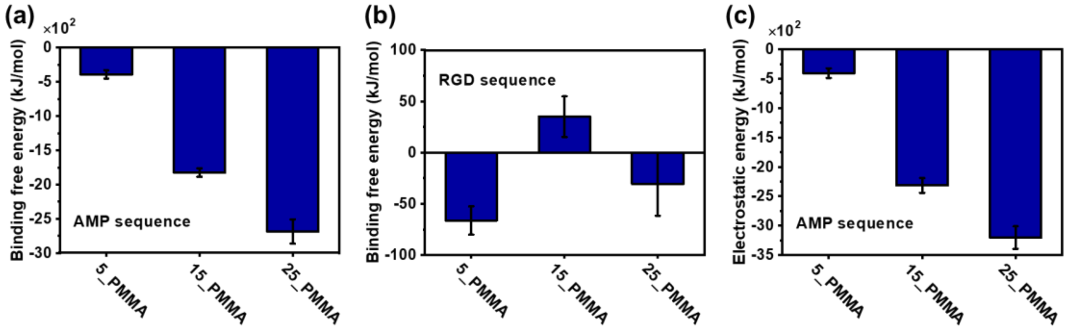

Figure S14 The binding free energy obtained from MM/PBSA method: (a) the binding free energy between the indicated surface and the AMP sequence in KD14; (b) the binding free energy between the indicated surface and the RGD sequence in KD14; (c) Contributions of electrostatic interactions to binding free energy between the indicated surface and the AMP sequence in KD14. The dielectric constant was 2 (ε=2). The error bars were estimated by the bootstrapping method[18]. Specifically, we bootstrapped 20 times, and at each time 40 MD conformations were randomly chosen. See Sec.9 and Sec.10 for details of the MM/PBSA calculations.

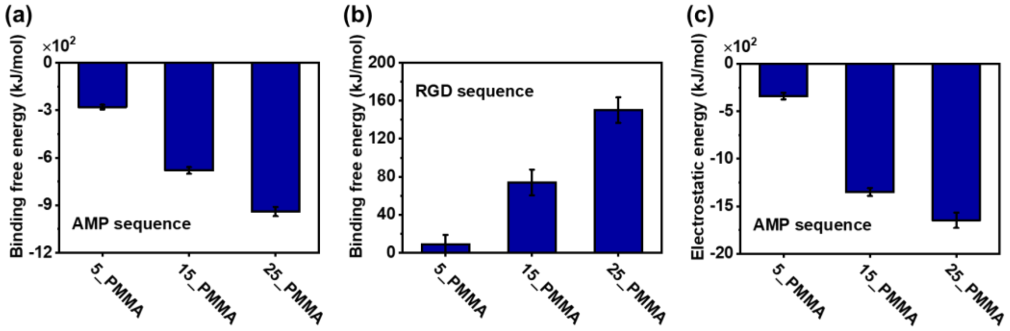

Figure S15 The binding free energy obtained from MM/PBSA method: (a) the binding free energy between the indicated surface and the AMP sequence in KD17; (b) the binding free energy between the indicated surface and the RGD sequence in KD17; (c) Contributions of electrostatic interactions to binding free energy between the indicated surface and the AMP sequence in KD17. The dielectric constant was 2 (ε=2). The error bars were estimated by the bootstrapping method[18]. Specifically, we bootstrapped 20 times, and at each time 40 MD conformations were randomly chosen. See Sec.9 and Sec.10 for details of the MM/PBSA calculations.

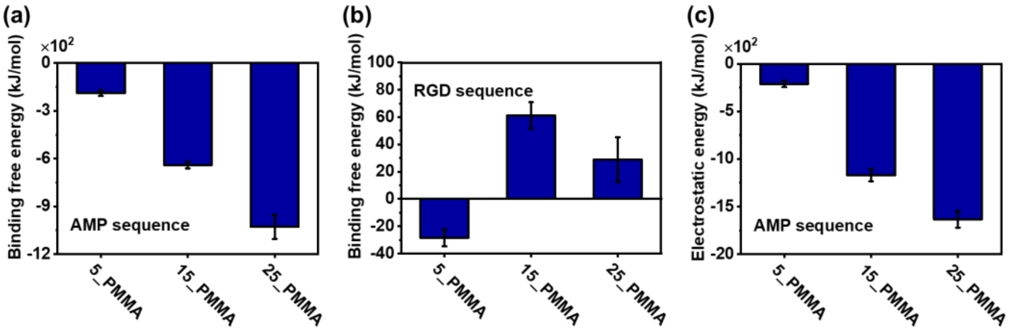

Figure S16 The CCK-8 assay of the *mBMSCs* on the indicated surface with HHC36. The optical density at 450 nm (OD_{450}) of the CCK-8 solution was analyzed to determine the viability of the cells. See Sec.7 for details of the CCK-8 assay.

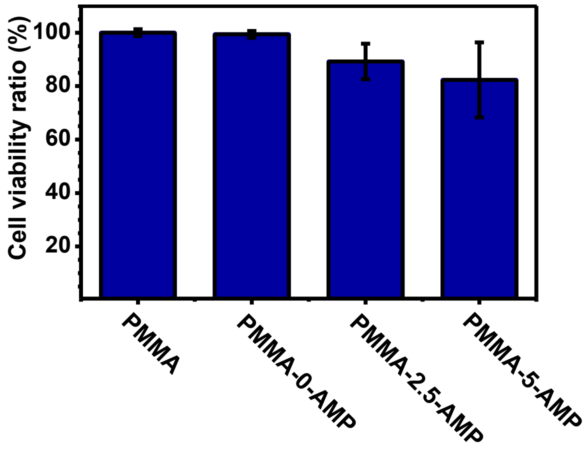

Figure S17 The fluorescent images of the *mBMSCs* on the indicated surfaces. The cells were stained with FITC and the images were obtained under the FITC channel of the confocal microscopy. See Sec.7 for details of the confocal microscopy assay. Scale bar = 200 μm.

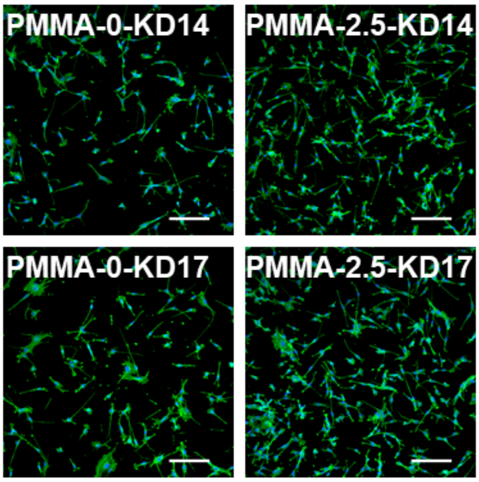

Figure S18 The antibacterial activity of the indicated surface with HHC36 against *S. aureus* and *E. coli* by the agar plate assay. See Sec.8 for details of the antibacterial assay.

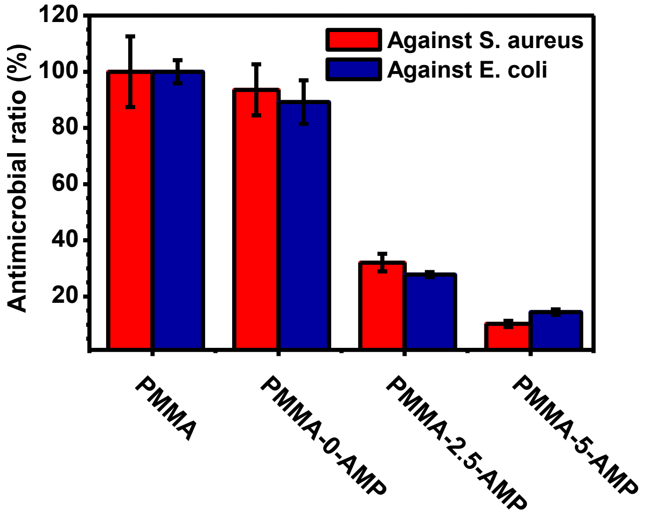

Figure S19 (a)The high-performance liquid chromatography and (b) the high resolution mass spectrometry results of CtrlP. See Sec.2 for details of the assay.

(a)

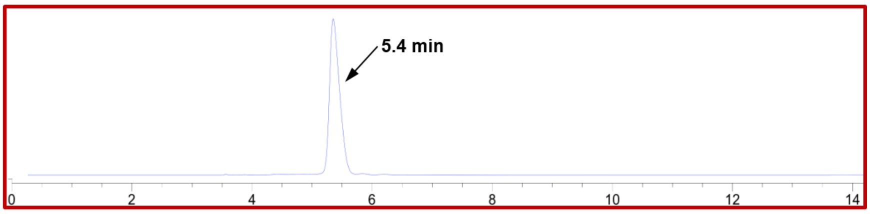

(b)

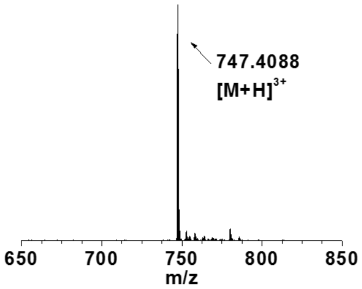

Figure S20 (a)The high-performance liquid chromatography and (b) the high resolution mass spectrometry results of CtrlP incubated with gelatinase for 1 h. See Sec.2 for details of the assay.

(a)

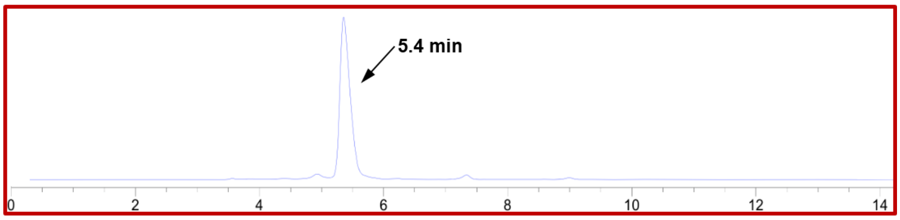

(b)

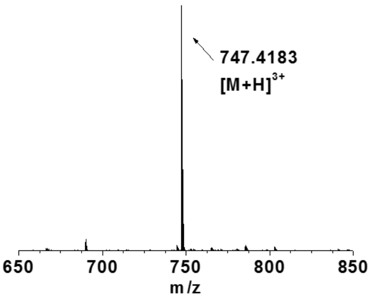

Figure S21 The CCK-8 assay of the *mBMSCs* on the indicated surface with CtrlP. The optical density at 450 nm (OD_{450}) of the CCK-8 solution was analyzed to determine the viability of the cells. See Sec.7 for details of the CCK-8 assay.

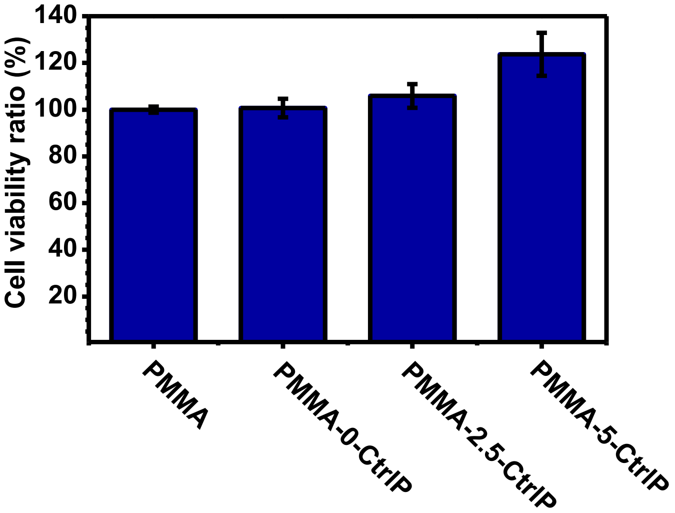

Figure S22 The antibacterial activity of the indicated surface with CtrlP against *S. aureus* and *E. coli* by the agar plate assay. See Sec.8 for details of the antibacterial assay.

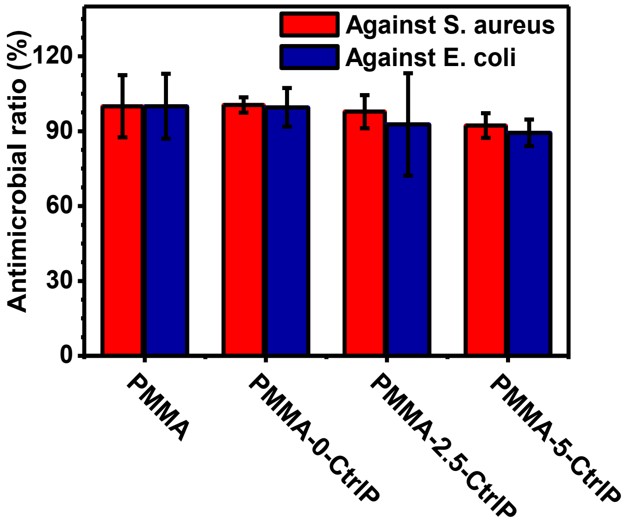

Figure S23 The bacterial accessible surface area (BASA) of the AMP sequence in KD14 and KD17 with different radii of the probes: (a) the radius of the probe was 0.14 nm, which was consistent of traditional probe in SASA; (b) the radius of the probe was 0.60 nm; (c) the radius of the probe was 1.80 nm. The results were calculated from 400 conformations extracted from the last 80 ns of each MD trajectory, in which the peptide has bound to the surface. See Sec.9 and Sec.10 for details of g_sas calculations.

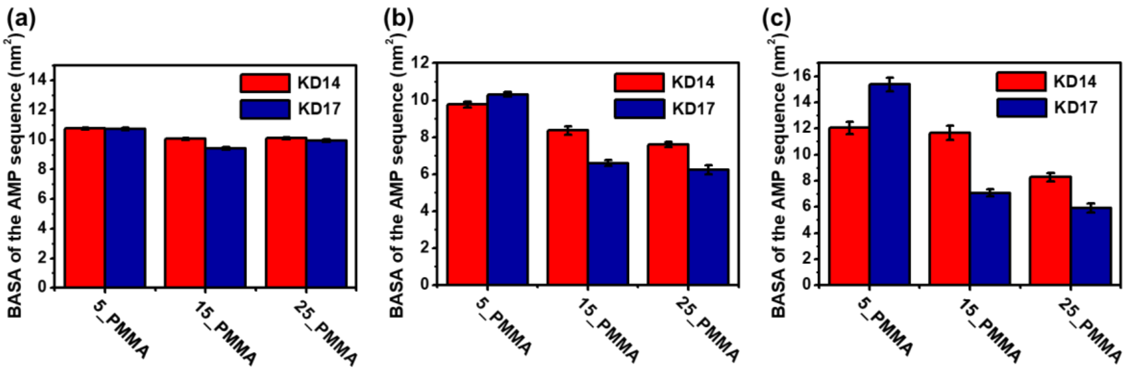

Figure S24 Projection of the conformations of peptide onto the radius of gyration and the distances of the C-N terminal of KD17 and KD14 on the surfaces with different charge densities. See Sec.9 and Sec.10 for details of these calculations.

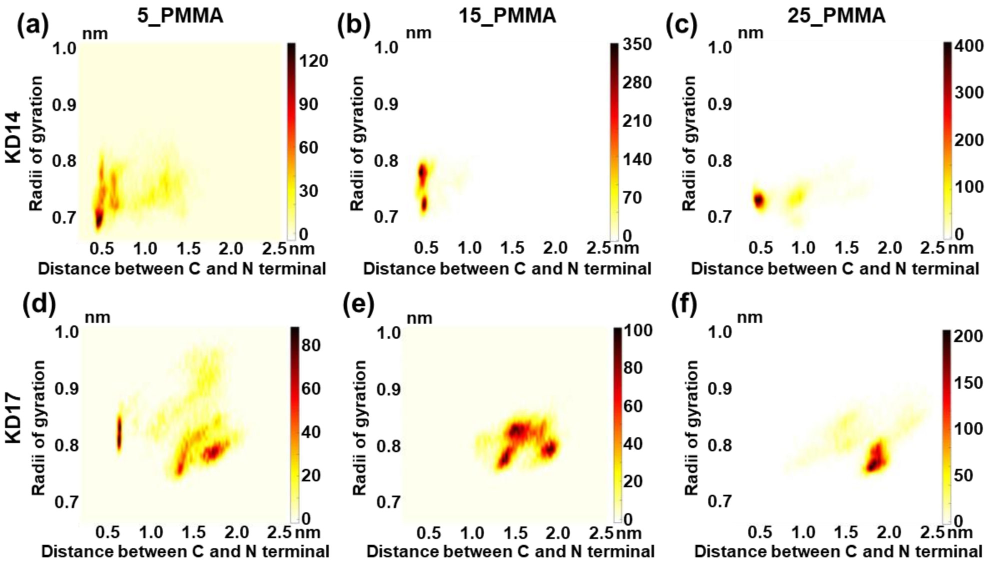

Figure S25 The antibacterial activity of the indicated surface *in vivo*. See Sec.11 for details of the *in vivo* assay.

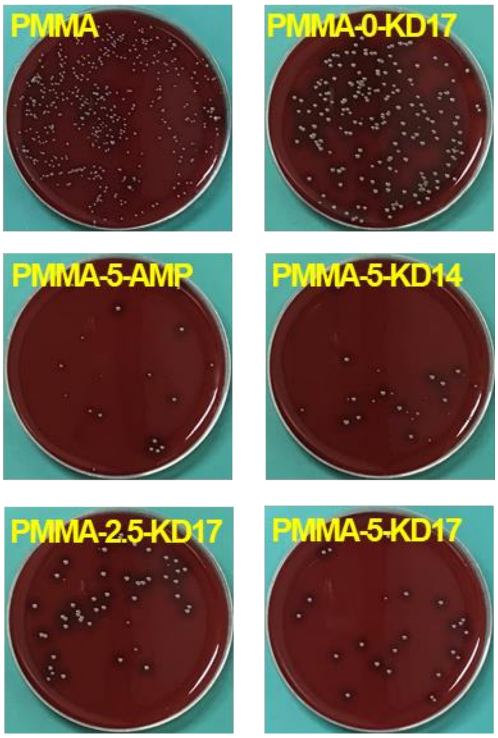

Table S1 The zeta potentials of the AMP sequence, RGD sequence, KD14 and KD17. See Sec.6 for details of the zeta potentials assay.

Samples	AMP sequence	RGD sequence	KD14	KD17
Zeta Potential (mV)	+14.1	-9.9	+5.8	+5.1

Table S2 The abbreviations of the samples.

Sample Abbreviation	Treatment Method
PMMA	Pristine PMMA substrate
PMMA-0min	PMMA treated with 0 min oxygen plasma
PMMA-2.5min	PMMA treated with 2.5 min oxygen plasma
PMMA-5min	PMMA treated with 5 min oxygen plasma
PMMA-0-AMP	PMMA-0min immersed into 500 μM of HHC36 solution
PMMA-2.5-AMP	PMMA-2.5min immersed into 500 μM of HHC36 solution
PMMA-5-AMP	PMMA-5min immersed into 500 μM of HHC36 solution
PMMA-0-KD14	PMMA-0min immersed into 500 μM of KD14 solution
PMMA-2.5-KD14	PMMA-2.5min immersed into 500 μM of KD14 solution
PMMA-5-KD14	PMMA-5min immersed into 500 μM of KD14 solution
PMMA-0-KD17	PMMA-0min immersed into 500 μM of KD17 solution
PMMA-2.5-KD17	PMMA-2.5min immersed into 500 μM of KD17 solution
PMMA-5-KD17	PMMA-5min immersed into 500 μM of KD17 solution
PMMA-0-CtrlP	PMMA-0min immersed into 500 μM of CtrlP solution
PMMA-2.5-CtrlP	PMMA-2.5min immersed into 500 μM of CtrlP solution
PMMA-5-CtrlP	PMMA-5min immersed into 500 μM of CtrlP solution

Table S3 The mass of KD14 and KD17 on different surfaces calculated by Q-tools from QCM-D results. See Sec.5 for details of the QCM-D assay.

	PMMA-0 (ng/cm^2)	PMMA-2.5 (ng/cm^2)	PMMA-5 (ng/cm^2)
KD14	46.5	136.1	238.5
KD17	5.0	62.2	136.6
